# Perineal Rectosigmoidectomy (Altemeier’s Procedure) in the Treatment of Strangulated Rectal Prolapse: A Case Series and Literature Review

**DOI:** 10.3390/jpm14111095

**Published:** 2024-11-06

**Authors:** Ioannis Mantzoros, Aliki Brenta, Aikaterini-Antonia Bourtzinakou, Ourania Kontaxi, Georgios Gemousakakis, Nikolaos Antoniou, Stefanos Bitsianis, Efstathios Kotidis, Dimitrios Kyziridis, Orestis Ioannidis, Ourania Kerasidou, Anna Gkiouliava, Manousos Pramateftakis, Stamatios Aggelopoulos

**Affiliations:** 1 4th Department of Surgery, General Hospital of Thessaloniki “G. Papanikolaou”, School of Medicine, Faculty of Health Science, Aristotle University of Thessaloniki, 541 24 Thessaloniki, Greece; imantzoros@auth.gr (I.M.); katerbourtzi@gmail.com (A.-A.B.); ourania.kontaxi@nhs.net (O.K.); ggemous@auth.gr (G.G.); sbits@auth.gr (S.B.); skotidis@auth.gr (E.K.); dkyzir@auth.gr (D.K.); iorestis@auth.gr (O.I.); koura@auth.gr (O.K.); mpramate@auth.gr (M.P.); saggelopoulos@auth.gr (S.A.); 2 2nd Department of Surgery, General Hospital of Thessaloniki “G. Gennimatas”, School of Medicine, Faculty of Health Science, Aristotle University of Thessaloniki, 541 24 Thessaloniki, Greece; nantoniou@auth.gr; 3 Department of Anesthesiology and Intensive Care, School of Medicine, Faculty of Health Science, Aristotle University of Thessaloniki, 541 24 Thessaloniki, Greece; annagkio@gmail.com

**Keywords:** rectal prolapse, incarcerated, perineal rectosigmoidectomy, Altemeier’s procedure

## Abstract

Background: Rectal prolapse (RP) predominantly affects women over the age of 50 and presents as mucosal, internal, or full thickness prolapse. Strangulated rectal prolapse requires immediate medical intervention, and surgical treatment options include both abdominal and perineal approaches. We aim to present a case series of perineal rectosigmoidectomy performed urgently due to strangulation and argue that Altemeier’s procedure is the preferred method for treating strangulated rectal prolapse. Methods: Perineal rectosigmoidectomy, particularly Altemeier’s procedure, is effective for incarcerated cases. Altemeier’s procedure with diverting ileostomy was used in all three patients. Results: All patients were successfully treated, with no recurrence of prolapse and stool incontinence. Conclusions: Altemeier’s procedure is ideal for the treatment of strangulated rectal prolapse.

## 1. Introduction

Rectal prolapse is an uncommon condition that occurs more frequently in women after the fifth decade. A strangulated (or incarcerated) rectal prolapse refers to a condition in which the full thickness prolapsing rectum cannot be manually repositioned. Unlike reducible prolapse, which can be pushed back into place, a strangulated prolapse remains extended, leading to mucosal or full thickness ischemia and resulting in septic complications. This condition requires immediate surgical intervention [1,2]. Surgical treatment options include both abdominal and perineal approaches [3]. Altemeier’s procedure is considered the preferred technique for treating strangulated rectal prolapse [1,2,3]. Here, we aim to present three cases of strangulated rectal prolapse treated at our hospital with Altemeier’s procedure, along with a literature review.

## 2. Description of Surgical Technique

Rectal prolapse can manifest clinically as mucosal prolapse (partial or pseudoprolapse), internal prolapse (rectal intussusception), or full thickness prolapse.

Various techniques have been proposed for the treatment of rectal prolapse, including abdominal approaches (Ripstein, Wells, and Orr-Loygue) and perineal procedures (Altemeier, Delorme, and Thiersch). Additionally, laparoscopic and open techniques have been developed, with or without the use of mesh. The choice of surgical method should be tailored to each patient’s specific condition and the extent of rectal prolapse. Perineal procedures are often performed under regional anesthesia. They are associated with lower morbidity and mortality, although they may have higher recurrence rates. Altemeier’s procedure typically involves perineal rectosigmoidectomy combined with anterior levatorplasty, which addresses the separation of the levator muscles seen in this condition, theoretically improving fecal continence [3,4,5]. Preoperatively, the large intestine is mechanically cleansed one day one day before surgery. The patient is positioned in the prone jackknife or Lloyd-Davies position, and a Foley catheter is inserted immediately after caudal anesthesia. Patients receive routine antibiotic prophylaxis.

During surgery, the prolapsed rectal mucosa is gradually grasped with Babcock or Allis clamps until the full-thickness prolapse is exposed, with eversion of the dental line. A circumferential incision is made 1.5 cm above the dentate line to achieve tissue dissection. The anterior peritoneal reflection is then incised to access the peritoneal cavity. The mesentery of the rectum and sigmoid colon is systematically clamped and ligated, and the mesenteric vessels are coagulated before removing the excess bowel. Optionally, the surgeon may perform anterior and posterior levatorplasty using separate 2-0 non-absorbable sutures to address the levator ani diastasis. The colon is then excised, and a coloanal anastomosis is created using interrupted sutures or staples. A protective ileostomy may also be performed, depending on the viability of the anastomosis.

## 3. Case Report

**Figure 1:**
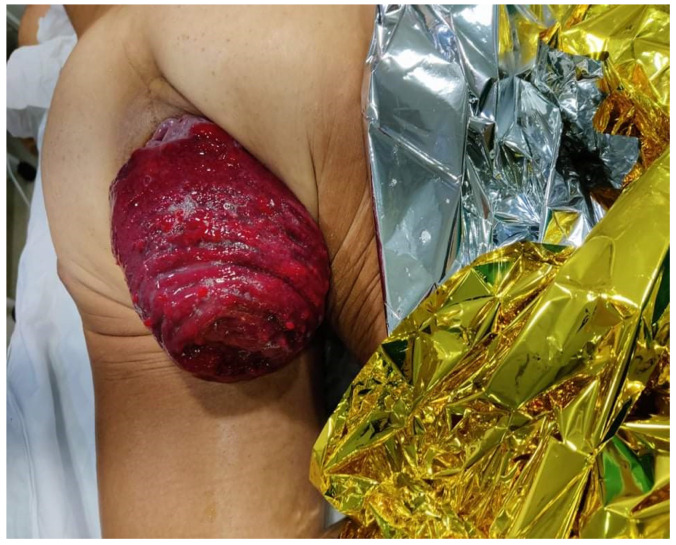
A 62-year-old male with incarcerated rectal prolapse with mucosal necrosis.

We present three cases of strangulated rectal prolapse using Altemeier’s procedure in an emergency setting:

Case 1: A 62-year-old male, a long-distance swimmer, presented with severe pain and a full-thickness incarcerated prolapse, showing signs of ischemia (Figure 1). Symptoms had begun earlier that day. The patient had a history of three previous hemorrhoidectomies and a lateral internal sphincterotomy for an anal fissure. The prolapse occurred five hours prior during defecation. An Altemeier procedure with a prophylactic ileostomy was performed under epidural anesthesia. The postoperative course was uneventful (Clavien–Dindo Classification: Grade 0), and the patient was discharged five days later. Ileostomy reversal was performed six months later. No levatorplasty was performed.

**Figure 2:**
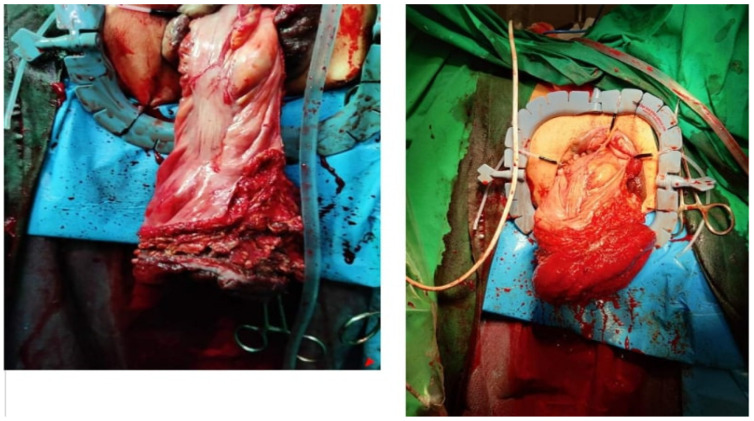
An 83-year-old female with large incarcerated prolapse.

Case 2: An 83-year-old female presented with a 20 cm full-thickness rectal prolapse, which she had been managing for the past five years. Initially, the prolapse was mechanically reducible, but over time, it progressively increased in length and became strangulated eight hours before admission (Figure 2). The patient had a history of three vaginal deliveries. Inflammatory markers were within normal range. Conservative treatments, including positioning the patient in the Trendelenburg position and applying granulated sugar topically, were attempted but unsuccessful. A rectosigmoidectomy was required, and an Altemeier procedure with a prophylactic ileostomy was performed. She was discharged seven days later with no postoperative complications (Clavien–Dindo Classification: Grade 0). No levatorplasty was performed.

**Figure 3:**
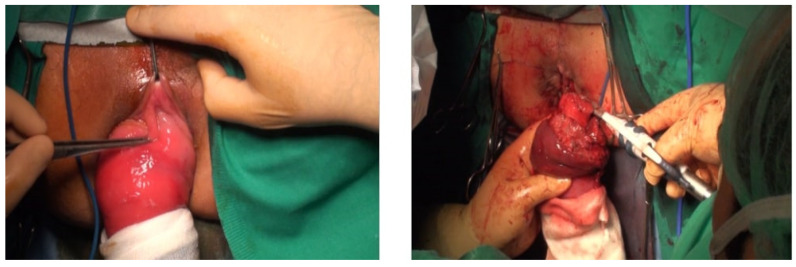
A 43-year-old female with a psychiatric background with pain and an incarcerated rectal prolapse.

Case 3: A 43-year-old female with a prolonged history of psychiatric institutionalization presented with pain and an incarcerated rectal prolapse that had worsened over the previous five days (Figure 3). The patient had a history of chronic constipation but no pregnancies. Altemeier’s procedure with a prophylactic ileostomy was performed under epidural anesthesia. On the fourth postoperative day, the patient developed sepsis due to aspiration pneumonia, presenting with fever and abnormal auscultatory findings. She recovered with antibiotic treatment (Clavien–Dindo Classification: Grade II) and was discharged on the fifteenth postoperative day. No levatorplasty was performed.

## 4. Follow-Up

Patients were scheduled for follow-up visits at 2 weeks and 3, 6, and 12 months after ileostomy reversal to ensure proper monitoring. Additional follow-up visits were scheduled at 24 and 36 months. Prolapse recurrence was assessed clinically, and incontinence was measured using the Cleveland Clinic Incontinence Score after ileostomy reversal. All three patients reported complete continence within the first 6 months after surgery. None of the patients experienced a recurrence of prolapse or stool incontinence during the follow-up period. To prevent incontinence, we advised all patients to perform Kegel pelvic floor exercises three times daily after undergoing Altemeier’s procedure.

## 5. Discussion

The prevalence of external rectal prolapse in the general population is less than 0.5%, with the majority of patients being women over the age of 50 [6]. Although the exact etiology of rectal prolapse is unknown, the most widely accepted theory, supported by defecography studies, is rectoanal intussusception [2]. Several factors appear to contribute, including multiple childbirths, the deterioration of the rectoanal inhibitory reflex, intermittent high-pressure rectal motor activity, anorectal sensory disorders, and pudendal neuropathy [1]. Rectal prolapse is more common among elderly patients, those with a history of vaginal births, chronic psychiatric disorders, and Ehlers–Danlos syndrome type 2. The literature associates rectal prolapse with age, multiparity, pelvic floor dysfunction, and perineal injury. Anatomical abnormalities, such as loose attachment of the rectum to the sacrum, lax lateral ligaments, a redundant sigmoid colon, a patulous anus, and diastasis of the levator ani muscles, are also linked to the development of rectal prolapse [3].

Initially, the prolapse may descend during defecation or straining but retract spontaneously afterward. Patients often report feeling a mass or lump that needs to be manually repositioned after bowel movements. Some may experience fecal incontinence caused by the prolapse or a persistent sensation of moisture and mucus discharge in the perineal area. A minor or spontaneously retractable prolapse can eventually progress to a chronically prolapsed rectum, requiring manual reduction. Over time, the rectal mucosa can become thickened or ulcerated, leading to significant bleeding. In rare cases, the prolapsed segment may become trapped below the anal sphincter, necessitating urgent surgical intervention.

According to the latest WSES-AAST (World Society of Emergency Medicine-American Association for the Surgery of Trauma) guidelines, the initial management of uncomplicated rectal prolapse involves non-operative methods, such as submucosal injection of epinephrine or hyaluronidase, rectal insertion of hypertonic dextrose- or mannitol-treated gauze, and elastic band wrapping, typically performed in a mildly sedated patient positioned in the Trendelenburg position. Strangulation of rectal prolapse is an uncommon complication, occurring in 2–4% of cases. In such instances, or when complications like hemodynamic instability, peritonitis, gangrene, or perforation arise, prompt surgical intervention through either perineal or abdominal approaches is recommended [4].

Various techniques exist for rectal prolapse repair, including abdominal and perineal approaches. Perineal surgery is associated with a higher recurrence rate, while abdominal surgery has a lower recurrence rate but a higher mortality risk and may be associated with complications such as impotence and infertility [5]. Abdominal approaches include suture rectopexy with or without resection, posterior mesh rectopexy, anterior sling (Ripstein procedure) rectopexy, and laparoscopic rectopexy [2]. Surgical options are more challenging in cases of incarceration due to the increased risk of anastomotic complications from bowel edema [6]. In cases where the bowel is irreducible and ischemic, excision may be required, which is typically achieved through a perineal approach [7]. The goal of surgery is to excise the affected bowel and any redundant tissue to prevent recurrence, although this can be difficult to assess, particularly when dealing with thickened, edematous tissues in strangulated cases.

The two preferred techniques for managing strangulated prolapse are mucosal resection with muscular plication (Delorme) and perineal rectosigmoidectomy (Altemeier), with younger, healthier individuals potentially benefiting from a transabdominal approach. Delorme’s procedure is suitable when ischemia is confined to the mucosa. In our series, all the patients presented with strangulated full-thickness rectal prolapse. In these cases, Delorme’s procedure, although less risky in older adult patients, is not problem-solving. For rectal prolapse repair, a perineal approach is generally favored over abdominal surgery, particularly for older or medically complex patients, as it results in fewer postoperative complications. One key advantage of this method is that it can be performed under epidural anesthesia, making Altemeier’s procedure especially appropriate for older patients with significant comorbidities. Ramanujan and Venkatesh underscored the effectiveness of perineal rectosigmoidectomy by successfully treating 8 older adult women among 70 cases over an 8-year period [8,9]. Despite its higher recurrence rate, the perineal approach is often preferred in older patients with comorbidities due to its lower complication rates and better patient tolerance. It is also a viable option in irreducible cases requiring emergency surgery.

Postoperative morbidity following perineal rectosigmoidectomy has been reported to be low, with complications occurring in only 10–12% of cases. The most serious complication, anastomotic leak, is more common in cases of incarcerated prolapse due to the presence of edema, which complicates anastomosis and increases the risk of breakdown. The risk of anastomotic leak in elective rectosigmoidectomy is 2–6%, but this rises to 25% in cases of incarceration [2]. Factors such as inadequate perfusion, edema, tension, and elevated intraluminal pressure contribute to the risk of anastomotic leaks, with an incidence of 10–15% in high-risk areas like the rectum and 5–10% elsewhere [10,11]. While fecal diversion can reduce the risk of leak, it does not eliminate it entirely.

Two recent studies found partial anastomotic dehiscence and leaks in a small number of patients undergoing perineal rectosigmoidectomy with or without diversion [12,13]. A case series reported two patients with incarcerated prolapse, with one requiring diversion due to concerns about mucosal necrosis; both had uneventful post-operative courses [6]. This suggests that anastomotic leaks are multifactorial and may still occur despite diversion [14,15,16], Thus, the decision to use adjunctive ileostomy should be customized according to the patient’s individual condition, overall health, and the specific circumstances of the prolapse. In our case series, a diverting loop ileostomy was performed in all three cases, and none of the patients experienced dehiscence. We used a trephine loop ileostomy technique, accessing the abdominal cavity through a small incision, which avoided the need for a full laparotomy. This approach is minimally invasive, and spinal anesthesia was sufficient. Spinal anesthesia was preferred, as it avoids further strain on already compromised patients, especially given the presence of bowel strangulation and serious comorbidities. Even when ileostomies are performed under general anesthesia, spinal anesthesia helps with postoperative pain management. Thus, the ability to perform this perineal technique under spinal anesthesia remains advantageous. The decision for stool diversion was particularly important considering the patients’ poor nutritional status, as the procedure was performed on an emergency basis in older adult patients with nutritional and psychiatric challenges. Moreover, this method facilitates the subsequent reversal of the ileostomy and the creation of an ileo-colonic anastomosis at a later stage.

Stool continence is another critical factor. Our patients reported intermittent incontinence due to constant bowel irritation before surgery. Stool incontinence in rectal prolapse results from the disruption and weakening of the anal sphincters and pelvic floor muscles, as well as nerve damage and irritation from chronic prolapse. These factors impair the ability to control stool passage and maintain continence. In all cases, continence was restored within 3 to 6 months after the procedure.

Many studies have evaluated continence following elective Altemeier’s procedure. The results of these studies are presented in Table 1. For this reason, perineal rectosigmoidectomy has been modified with the addition of levatorplasty. The main reasons for adding levatorplasty are to address the high preoperative rate of fecal incontinence and to prevent recurrent prolapse. Chun et al. reported recurrence rates of 7.7% with levatorplasty compared with 20.6% without it [17]. This procedure improves anal continence by restoring the anorectal angle and offers additional benefits, such as lower short-term recurrence rates and a longer recurrence-free interval. However, other studies suggest that levatorplasty may not add significant value when combined with Altemeier’s procedure [18,19,20,21]. As a team, we opted not to perform levatorplasty in some cases because the levator ani muscle is often already weakened, and attempts to restore it might further compromise its integrity, potentially worsening incontinence. Bananzadeh et al. compared the outcomes of Altemeier’s procedure performed with and without posterior levatorplasty [22]. In their retrospective study, they found no significant reduction in fecal incontinence with posterior levatorplasty. The pathophysiology of incontinence related to rectal prolapse involves pelvic muscle relaxation, pudendal nerve paralysis, and sphincter relaxation. Therefore, we believe that postoperative muscle training, biofeedback, and nerve stimulation may be more beneficial than levatorplasty in improving patient outcomes. These treatments have been used successfully for incontinence improvement in the past [23,24,25].

The relationship between sphincterotomy in one of our patients and rectal prolapse is not definitively proven. The internal sphincter contributes approximately 25–30% to the continence mechanism [19,20]. In the first case, in which the patient had previously undergone internal sphincterotomy, attempting to surgically restore the internal sphincter’s anatomy would likely not have significantly affected the patient’s continence. Our approach is to follow up postoperatively and, in cases of severe incontinence, assess the sphincter mechanism using manometry, endoscopic ultrasound, and MRI, and then decide whether anatomical restoration is warranted. The authors suggest that internal sphincter restoration would not benefit neither the continence nor the recurrence rates.

Recurrence rates are generally higher with the perineal approach (up to 20% after Altemeier’s procedure and up to 30% after Delorme’s) than with the abdominal approach [16]. According to the literature [7], several technical factors play a significant role. Kim et al. found that patients who underwent stapled anastomosis had a significantly higher recurrence rate than those with hand-sewn anastomosis [26]. Additionally, patients with a shorter length of resected tissue were at greater risk of relapse than those with longer resections. Furthermore, having a prolapse for more than six months prior to surgery was associated with a higher risk of recurrence, although this increase was not statistically significant [26]. Several studies indicate that recurrence is linked to factors such as the length of bowel resected, the surgeon’s level of experience, and the type of anastomosis performed [19,27,28]. In our opinion, attempting to repair the internal sphincter may compromise the sphincter mechanism more than it would benefit continence or reduce recurrence. While the literature identifies various factors contributing to recurrence, many of these are unrelated to the sphincter mechanism. Thus, we believe that repairing the internal sphincter would not significantly reduce the risk of recurrence. Recurrence following Altemeier’s procedure typically depends on technical fouls, and the majority of recurrences present in the first year following the operation. The most common cause of recurrence is excision of inadequate sigmoid resection. In these cases, the spare mobile sigmoid colon prolapses again and results in early recurrence. In our series, the follow-up was 36 months, which is considered a sufficiently safe time frame to make conclusions about recurrences related or unrelated to technical errors.

All techniques for the restoration of rectal prolapse, whether perineal or abdominal, carry recurrence rates ranging between 0–30% [16,29,30]. Altemeier’s procedure does not directly affect the internal sphincter or pelvic floor muscles, but it can alleviate constipation by removing the redundant sigmoid colon. However, the underlying causes of prolapse often remain, and perineal techniques are generally associated with higher—though acceptable—recurrence rates. The literature links recurrence more to technical factors than physiological ones [19,27,28]. It is important to note that our case series involves strangulated rectal prolapse with ischemic bowel, which made the abdominal approach unsuitable. Our primary goal was to salvage the remaining colon, as Altemeier’s procedure was performed as an emergency. In this context, recurrence was a secondary concern.

Incorporating laparoscopy into the perineal approach for rectal prolapse is an innovative technique. It aids in evaluating the length of the sigmoid, facilitates colonic mobilization, and ensures thorough excision of redundant bowel. By directly assessing bowel length and redundancy, laparoscopy helps the surgeon determine the necessary extent of resection [15]. However, laparoscopy is contraindicated in cases of strangulation, as attempting to reduce ischemic or necrotic bowel back into the abdominal cavity can lead to infection spread and sepsis due to compromised tissue.

Most of the articles published regarding Altemeier’s procedure, as shown in Table 1, either do not specify the type of prolapse (whether chronic or acutely complicated by incarceration and strangulation) or refer to elective surgeries. In the context of incarcerated prolapse, surgical treatment options are extremely limited, and the literature suggests that the ideal approach is Altemeier’s procedure [8,31]. Altemeier’s procedure is typically preferred for patients with comorbidities because it can be performed under spinal or regional anesthesia, avoiding the need for laparotomy and allowing for quicker recovery and earlier mobilization [21]. Altemeier’s procedure is typically preferred for patients with comorbidities because it can be performed under spinal or regional anesthesia, avoiding the need for laparotomy and allowing for quicker recovery and earlier mobilization [18,21,27,32,33]. By contrast, abdominal repair requires general anesthesia and poses the risk of pelvic adhesions, which may compromise fertility in young women and cause impotence in men. There is also the risk of anastomotic leakage if a resection rectopexy is performed, though resections are now rarely performed [34]. The minimally invasive nature of Altemeier’s procedure and its ability to be repeated without significantly increasing the risk of complications make it an ideal choice for patients with strangulated prolapse and those with comorbidities [21].

Most studies focus on patients undergoing rectosigmoidectomy in an elective setting, as strangulation is a rare complication. When the prolapsed bowel becomes incarcerated or gangrenous and cannot be repositioned using standard methods, surgical intervention becomes an emergency, as was the case for our patients. Immediate surgery is crucial to prevent compromising bowel viability, as gangrene significantly increases the risk of complications and mortality [5]. Altemeier’s procedure remains the gold standard for treating incarcerated prolapse, surpassing abdominal approaches since necrotic bowel cannot be returned to the abdominal cavity and requires full-thickness resection. Additionally, the perineal approach avoids the use of mesh, reducing the risk of infection at a new site. As noted earlier, perineal procedures are particularly suitable for high-risk patients. An abdominal surgery to remove the affected segment would likely require a colostomy and would carry higher risks for these already compromised patients.

Apart from those presented in Table 1, there are several case reports in the literature regarding the treatment of strangulated prolapse using Altemeier’s technique. Voulimeneas et al. reported a complicated case of gangrenous rectal prolapse treated with Altemeier’s procedure and prophylactic ileostomy [5], while Nguyen et al. successfully treated two cases of irreducible incarcerated prolapse with perineal rectosigmoidectomy, one of which required fecal diversion [35]. Cernuda et al. found no recurrence of prolapse six months after performing Altemeier’s procedure with loop ileostomy [9]. Recent case reports also demonstrate positive outcomes in treating strangulated rectal prolapse using Altemeier’s technique, further underscoring its effectiveness [36,37,38].

Our study, while limited by its retrospective design and small sample size, addresses the rare emergency of rectal prolapse complicated by strangulation. The design of an RCT or a meta-analysis is difficult, considering the rarity of strangulated cases. The available literature on this condition is sparse, and most studies in our literature review focus on elective Altemeier’s procedures. For this reason, our sample is very specific, and to our knowledge, there are no systematic reviews or larger studies regarding the management of strangulated rectal prolapse specifically. Furthermore, since our cases involved strangulated prolapse, abdominal procedures were not a viable option, precluding a comparison of approaches. When strangulation and ischemic bowel are present, there is an indication for Altemeier’s technique [1,2,5,6,7,8,9,31]. The goal of this study was to present the outcomes of our case series involving three patients who underwent urgent Altemeier’s procedures due to strangulation. There is limited evidence regarding outcomes with other techniques in cases of strangulation, but individual case reports support our view that Altemeier’s technique is the ideal treatment for this rare emergency.

**Table 1:** Literature review of results after Altemeier’s Procedure.

Authors	N	Study	Levatorplasty	Incarcerated # (%)	Mortality # (%)	Continence %	Constipation %	Recurrence # (%)	Follow-Up, Months
Johansen et al., 1993 [39]	20	Not stated	No	No	1 (5)	21	Not stated	0	26
Ramanujam et al., 1994 [40]	72	Not stated	No	9 (13)	0	67	Not stated	4 (6)	120
Deen et al., 1994 [41]	10	Prospective	No	No	0	80	Not stated	1 (10)	18
Agachan et al., 1997 [29]	32	Retrospective	No	No	0	+	Not stated	4 (13)	30
Takesue et al., 1999 [31]	10	Not stated	Yes (7)	Not stated	0	+	Not stated	0	42
Kim et al. 1999 [28]	183	Retrospective	No	Not stated	Not stated	53	61 (+)	29 (16)	47
Kimmins et al. 2001 [21]	63	Retrospective	Yes (29)	4 (6)	0	87	31.7	4 (6.3)	21
Zbar et al., 2002 [42]	80	Retrospective	Yes	Not stated	0	100	Not stated	3 (3.8)	22
Chun et al., 2004 [17]	109	Retrospective	Yes	Not stated	0	100	Not stated	18 (16.5)	29
Habr gama et al., 2006 [43]	44	Retrospective	Yes	Not stated	0	85	Not stated	3 (7.1)	49
Boccasanta et al., 2006 [44]	40	RTC	Yes	Not stated	Not stated	100	Not stated	5 (12.5)	28
Altomare et al., 2009 [16]	93	Retrospective	No	Not stated	0	47	+	5 (25)	41
Cirocco WC 2010 [27]	103	Not stated	Yes	Not stated	0	85	94 (+)	0	43
Ris et al., 2011 [18]	60	Not stated	Yes	Not stated	1.6	62	Not stated	(14)	48
Senapati et al., 2013 [33]	102	RTC	Not stated	Not stated	(2)	(+)	Not stated	24 (24)	60
Kim et al., 2014 [26]	63	Retrospective	Yes (33)	Not stated	(1.6)	Not stated	Not stated	8 (12.7)	33
Trompeto et al., 2019 [45]	43	Retrospective	Yes (21)	Not stated	0	No change	(+)	(40)	49
Boccasanta et al., 2021 [30]	130	Retrospective	Yes	Not stated	4 (3.1)	(+)	(+)	27 (20.8)	82

#: absolute number; (+): favorable results but no specified percentage.

## 6. Conclusions

In conclusion, perineal rectosigmoidectomy stands as a viable treatment option for incarcerated and strangulated rectal prolapse, offering low mortality and acceptable recurrence rates. The literature, as well as our experience, emphasizes the importance of early surgical intervention, particularly in cases of strangulated rectal prolapse complicated by necrosis. This underscores the value of perineal proctosigmoidectomy in addressing this rare surgical emergency. Overall, Altemeier’s procedure is ideal for the treatment of strangulated rectal prolapse.

## Data Availability

No new data were created or analyzed in this study. Data sharing is not applicable to this article.
